# The Tryptophan-Rich Sensory Protein (TSPO) is Involved in Stress-Related and Light-Dependent Processes in the Cyanobacterium *Fremyella diplosiphon*

**DOI:** 10.3389/fmicb.2015.01393

**Published:** 2015-12-14

**Authors:** Andrea W. U. Busch, Beronda L. Montgomery

**Affiliations:** ^1^Department of Energy – Plant Research Laboratory, Michigan State University, East LansingMI, USA; ^2^Department of Biochemistry and Molecular Biology, Michigan State University, East LansingMI, USA

**Keywords:** cyanobacteria, light signaling, osmotic stress, oxidative stress, reactive oxygen species (ROS), salt stress, tryptophan-rich sensory protein (TSPO)

## Abstract

The tryptophan-rich sensory protein (TSPO) is a membrane protein, which is a member of the 18 kDa translocator protein/peripheral-type benzodiazepine receptor (MBR) family of proteins that is present in most organisms and is also referred to as Translocator protein 18 kDa. Although TSPO is associated with stress- and disease-related processes in organisms from bacteria to mammals, full elucidation of the functional role of the TSPO protein is lacking for most organisms in which it is found. In this study, we describe the regulation and function of a TSPO homolog in the cyanobacterium *Fremyella diplosiphon*, designated *Fd*TSPO. Accumulation of the *FdTSPO* transcript is upregulated by green light and in response to nutrient deficiency and stress. A *F. diplosiphon TSPO* deletion mutant (i.e., Δ*FdTSPO*) showed altered responses compared to the wild type (WT) strain under stress conditions, including salt treatment, osmotic stress, and induced oxidative stress. Under salt stress, the *FdTSPO* transcript is upregulated and a Δ*FdTSPO* mutant accumulates lower levels of reactive oxygen species (ROS) and displays increased growth compared to WT. In response to osmotic stress, *FdTSPO* transcript levels are upregulated and Δ*FdTSPO* mutant cells exhibit impaired growth compared to the WT. By comparison, methyl viologen-induced oxidative stress results in higher ROS levels in the Δ*FdTSPO* mutant compared to the WT strain. Taken together, our results provide support for the involvement of membrane-localized *Fd*TSPO in mediating cellular responses to stress in *F. diplosiphon* and represent detailed functional analysis of a cyanobacterial TSPO. This study advances our understanding of the functional roles of TSPO homologs *in vivo*.

## Introduction

Cyanobacteria are capable of adapting to various environments as evident by their ubiquitous distribution in fresh and salt water bodies as well as arid areas (Whitton and Potts, 2000). These organisms exhibit developmental plasticity in response to environmental cues such as light and/or nutrients to tune growth and development to their environment. The large peripheral light-harvesting antennae, the phycobilisomes (PBSs), that are found in cyanobacteria transfer absorbed light energy to the core photosystems to enable efficient light-harvesting under different light qualities and quantities (Grossman et al., 1993; Watanabe and Ikeuchi, 2013). The filamentous, freshwater cyanobacterium *Fremyella diplosiphon* is a model organism for complementary chromatic acclimation (CCA; Bennett and Bogorad, 1973; Kehoe and Grossman, 1994; Kehoe and Gutu, 2006). During CCA, the composition of PBSs is varied in this organism through transcriptional control of phycobiliprotein biosynthesis depending on the external light quality (Gutu and Kehoe, 2012). *F. diplosiphon* synthesizes two major phycobiliproteins that make up the external portion of the PBS rods, the green light- (GL) absorbing phycoerythrin (PE) and the red light- (RL) absorbing phycocyanin (PC; Takemoto and Bogorad, 1975; Conley et al., 1988). The phycobilin chromophores that confer the PBSs with their specific absorption characteristics are open-chain tetrapyrroles covalently attached to the phycobiliproteins (Frankenberg et al., 2001).

Tetrapyrroles are involved in many important processes in cells, including respiration, photosynthesis, and methanogenesis (Warren and Smith, 2009). Tetrapyrrole biosynthesis bifurcates into the heme branch and the chlorophyll branch after the formation of cyclic tetrapyrrole protoporphyrin IX (Warren and Smith, 2009). Heme biosynthesis requires chelation of iron, while its oxidative cleavage to form the open chain-tetrapyrroles, including those used to synthesize PBSs, involves iron release (Ferreira et al., 1995; Unno et al., 2007). Therefore, a tight regulation of tetrapyrrole synthesis and iron homeostasis is indispensable for regulating oxidative stress responses (Busch and Montgomery, 2015), especially due to the photosensitizing activity of tetrapyrroles (Aravind Menon et al., 1989) and the function of iron as a cofactor in fighting oxidative stress (Busch and Montgomery, 2015).

Responses to light, availability of iron and other nutrients, tetrapyrrole metabolism, and oxidative stress are co-regulated. For example, iron uptake and transport are light-regulated in photosynthetic organisms, often linked to photoreceptors (Montgomery et al., 2015). In *F. diplosiphon* the photoreceptor RcaE controls not only chromatic acclimation (Kehoe and Grossman, 1996; Terauchi et al., 2004), but is also involved in acclimation to iron-deficiency (Pattanaik et al., 2014). Degradation of light-harvesting PBSs as well as a restructuring of the photosynthetic apparatus can reduce oxidative stress under high-light exposure and/or nutrient deficiency (Narayan et al., 2011; Busch and Montgomery, 2015). Also, tetrapyrrole metabolism is largely controlled by light and involves feedback regulation by heme in photosynthetic organisms (Warren and Smith, 2009). Our aim was to identify factors involved in the process of integrating light and stress responses with pigment metabolism.

The tryptophan-rich outer membrane sensory protein (tryptophan-rich sensory protein, TSPO), more recently referred to as translocator protein 18 kDa (TSPO) although its role as a ubiquitous transporter is still under debate (Batoko et al., 2015), is an outer membrane protein in photosynthetic and non-photosynthetic organisms that appears to be involved in tetrapyrrole metabolism, stress adaptation and regulation of carotenoid biosynthesis (Yeliseev and Kaplan, 1995, 1999; Davey and de Bruijn, 2000; Papadopoulos et al., 2006; Guillaumot et al., 2009; Balsemão-Pires et al., 2011; Vanhee et al., 2011). TSPO was first identified and studied in mammals where it was previously called peripheral-type benzodiazepine receptor (Braestrup et al., 1977). Although present in various genomes throughout all kingdoms of life, including vertebrates, invertebrates, plants, some yeasts (i.e., found in *Schizosaccharomyces pombe*, but not in *Saccharomyces cerevisiae*) and bacteria, TSPO is not ubiquitous. It belongs to a family of membrane proteins (Pfam: TspO/MBR family PF03073) that bind a large variety of ligands and functions for TSPO in stress, photosynthesis, membrane transport, and human diseases all have been implicated (Yeliseev and Kaplan, 1999; Wendler et al., 2003; Balsemão-Pires et al., 2011; Colasanti et al., 2013; Wu and Gallo, 2013).

A *TSPO* homolog is expressed in *F. diplosiphon* (Luque et al., 2003; Stowe-Evans et al., 2004). *FdTSPO* expression was reported as increased in nitrogen-replete medium (Luque et al., 2003) or by GL (Stowe-Evans et al., 2004). To investigate the functional role of TSPO in responses to light and stress in *F. diplosiphon*, we produced a complete deletion of the *TSPO* homolog (Δ*FdTSPO*) in this organism. Comparative phenotypical analyses of Δ*FdTSPO* and wild type (WT) cells grown under salt stress demonstrated that the Δ*FdTSPO* mutant exhibited lower pigment content and decreased ROS levels, whereas growth was increased in the Δ*FdTSPO* mutant compared to WT under these conditions. Under osmotic stress conditions, the Δ*FdTSPO* mutant grew slower than WT. The salt and osmotic stress-dependent observations were light-quality dependent. Induction of oxidative stress by methyl viologen (MV) resulted in higher ROS levels in the mutant compared to the WT under GL. The Δ*FdTSPO* mutant also exhibited altered light-dependent regulation of cellular morphology compared to WT. Our results provide evidence that *Fd*TSPO functions during organismal responses to stress and exhibits distinct light-quality dependent roles in *F. diplosiphon*.

## Materials and Methods

### Bacterial Strains and Growth Conditions

*Fremyella diplosiphon* strain SF33, which is a short filament, wild-type pigmentation strain (Cobley et al., 1993), was used as the WT parent. *F. diplosiphon* strains were grown at 28 °C in BG-11 medium (Fluka, Buchs, Switzerland) with 20 mM HEPES (hereafter BG-11/HEPES) with shaking at 175 rpm at ∼10 μmol m^-2^ s^-1^ of continuous broad-band GL (CVG sleeved Rosco green 89 fluorescent tubes, General Electric; model no. F20T12/G78) or continuous broad-band RL (CVG sleeved Rosco red 24 fluorescent tubes, General Electric; model no. F20T12/R24). Light intensities were measured using an LI-250A light meter (LI-COR, Lincoln, NE) equipped with a quantum sensor (LI-COR). Cell densities were determined by measuring the optical density at 750 nm (OD_750_) using a SpectraMax M2 microplate reader (Molecular Devices, Sunnyvale, CA, USA). Iron-replete and -depleted cells were grown as described previously (Pattanaik et al., 2014). Salt-treated cells were grown in BG-11/HEPES medium supplemented with 200 mM NaCl at ∼10 μmol m^-2^ s^-1^. Osmotic stress treatment was accomplished by growing cells in BG-11/HEPES medium supplemented with 400 mM sorbitol at ∼10 μmol m^-2^ s^-1^.

*Escherichia coli* cultures were grown at 37°C in Luria–Bertani (LB) broth with the indicated antibiotic [i.e., 100 μg/ml (w/v) ampicillin, 50 μg/ml (w/v) kanamycin, or 10 μg/ml (w/v) neomycin]. For growth on solid medium 1.5% (w/v) Bacto-Agar in LB was used.

### Mutant Generation

A *FdTSPO* knock out mutant (i.e., Δ*FdTSPO*) was generated in the WT background. Allelic-exchange vector pJCF276 (Cobley et al., 2002) carrying ∼4 kb of the genomic region containing *FdTSPO* (GenBank accession number of corresponding protein: AAT36314.1) was constructed. The initial fragment was obtained by amplifying the *FdTSPO*-containing genomic region (*NcoI* restriction sites underlined; forward primer: cctccatgggcgtttagttatctggaaacc, reverse primer: gaaccatggccatcttacgcaatttgg) with Prime Star GXL polymerase (Clontech, Mountain View, CA, USA). The vector and insert were restricted with *NcoI* restriction enzyme and ligated with the TaKaRa DNA ligation kit version 2.1 (Clontech, Mountain View, CA, USA). The *FdTSPO* gene (i.e., 756 bp coding region) was then deleted from this construct by whole-plasmid PCR using the same polymerase and 5′ phosphorylated primers (forward primer: aattaagatattgagcttcgctggtaattattaataataaatcatcagc, reverse primer: atactcaaaattaattttgacatcaatagcagtagagaaattagcaatc) followed by ligation with the TaKaRa DNA ligation kit. The verified donor plasmid was transformed into DH5α MCR *E. coli* cells containing the plasmid pJCF173, which contains methylase genes to produce methylated plasmids that are protected from digestion in the *F. diplosiphon* host (Cobley et al., 1999). Fully segregated transconjugants were obtained essentially using the method described before (Elhai and Wolk, 1988; Cobley et al., 1993, 2002; Pattanaik and Montgomery, 2010). The first selection was carried out on neomycin-containing plates and the second selection was on 5% sucrose-containing medium. Full segregation was confirmed by comparative PCR using WT and putative mutant *F. diplosiphon* cells as templates. PCR was performed with primers located in the flanking region of *FdTSPO* (forward primer: caggtgggactggtcac, reverse primer: ttaacaaaagttacgcctgc), resulting in a product of 2208 and 1452 bp for the amplified region in WT or Δ*FdTSPO*, respectively.

### Production of Complemented Δ*FdTSPO* Strain

The gene for *FdTSPO* including its 360 bp upstream region was amplified with primers adding attB-sites for subsequent Gateway^®^ cloning (fwd primer: ggggacaagtttgtacaaaaaagcaggcttcggattgcaggtaagtagagc, reverse primer: ggggaccactttgtacaagaaagctgggtcttatttttccactggtgtgag). The resulting product was cloned into the pDONR^TM^/Zeo vector according to the manufacturer’s instructions. The resulting donor vector was then used in an LR recombination reaction with the pPL2.7-GWC vector containing a Gateway^®^ cassette (Bordowitz and Montgomery, 2008). An empty pPL2.7 vector (Cobley et al., 1993) and pPL2.7 containing *FdTSPO* with its native promoter (pPL2.7_npTSPO) were transformed into WT (SF33) *F. diplosiphon* cells by means of triparental mating essentially as described (Pattanaik and Montgomery, 2010). Selection was carried out on BG11/HEPES plates containing 25 μg/ml kanamycin.

### Growth Measurements

Cells adapted to the indicated light color at ∼10 μmol m^-2^ s^-1^ were diluted in exponential phase to an OD_750_ of 0.1 to initiate growth experiments. Cell density was measured as OD_750_ in two- or 3-day intervals. The growth was measured for at least three biological replicates. Growth curves were obtained for WT and Δ*FdTSPO* cells under controlled-environment conditions in GL or RL at fluence rates of ∼10 μmol m^-2^ s^-1^.

### ROS Measurements

Reactive oxygen species measurements were performed using a cell-permeable ROS-sensitive 2′, 7′-dichlorodihydrofluorescein diacetate (DCFH-DA) dye essentially as described before (Singh and Montgomery, 2012) with the following adjustments. Before every DCFH-DA addition, cells with an OD_750_ > 0.2 were diluted with growth medium to OD_750_ = 0.2 in 1 ml of medium.

Methyl viologen dichloride (Sigma–Aldrich, St.Louis, MO, USA) was added from a 1 mM (w/v) stock solution in ddH_2_O to a final concentration of 0.3 μM (v/v) in the liquid medium. Salt-treated cells were treated as described above. ROS content was measured before and 72 h after treatment with MV and after 6 days of growth in 200 mM salt.

### Microscopy-based Analysis of Cellular Morphology

Slide preparation and confocal laser scanning microscope imaging were conducted to determine cell size essentially as described previously (Bordowitz and Montgomery, 2008, 2010). Cells in the exponential phase were diluted to OD_750_ = 0.1 and grown in the respective light for an additional 3 days prior to imaging.

### Pigment Extraction and Quantification

Phycobiliproteins, chlorophyll *a* (chl*a*) and carotenoids were extracted and quantified as described (Tandeau de Marsac and Houmard, 1988; Kahn et al., 1997; Bordowitz and Montgomery, 2008), with minor modifications. A cell pellet equivalent to 1 ml of a culture at OD_750_ = 0.6 was harvested in mid- to late exponential phase. Phycobiliprotein extraction was performed for up to 90 min. Calculations were conducted as previously described (Dere et al., 1998; Bordowitz and Montgomery, 2008).

### Reverse Transcription Polymerase Chain Reaction

Wild type or Δ*FdTSPO* cells were grown in the respective light condition to exponential phase and diluted to OD_750_ = 0.6 in BG11/HEPES before being grown for an additional 16 h. For analysis of sorbitol treatment after dilution and growth for 16 h, a 10 ml sample was taken as the 0 h time point and the remaining culture was pelleted at 4°C at 4750 rpm for 10 min. The pellet was resuspended in 40 ml of BG11/HEPES containing 400 mM of sorbitol to initiate osmotic stress. For analysis of salt or MV treatment, after removing a 10 ml sample for the 0 h pre-treatment timepoint, NaCl was added to a final concentration of 200 mM (v/v) or MV was added to a final concentration of 0.3 μM (v/v). For all samples, i.e., untreated or treated, 10 or 50 ml of culture were harvested and RNA was extracted from the pellet with 1 ml Trizol per sample as described previously (Seib and Kehoe, 2002). Further RNA treatment and reverse transcription (RT) were performed as described before (Singh and Montgomery, 2013a). Primers used for reference gene *ORF10B* were those previously reported (Singh and Montgomery, 2013a). The following primers were used to detect *FdTSPO*: gtagaacggagattaggtgcg, forward primer; cagccacagttagccagatac, reverse primer. For analysis of *FdTSPO* abundance in the WT vs. mutant or for sorbitol or MV-treated cells, 500 ng of total RNA were used in a 20 μl RT reaction containing 1X buffer, 1.25 mM MgCl_2_, 1 mM dNTP mix, 0.5 μl RNasin, 1 μl random primers, and 0.7 μl AMVRT. A 10 μl qPCR reaction contained 5 μl Fast SYBR Green Master Mix, 0.4 μM primers, and 4 μl of a 1:40 dilution of the RT reaction using the Microamp^®^ fast optical 96-well reaction plate and ABI FAST 7500 Real-Time PCR system (Applied Biosystems, Grand Island, NY, USA) in FAST mode according to the manufacturer’s instructions. For analysis of salt treatment, 100 ng of total RNA were used in the RT reaction containing 1X buffer, 1.25 mM MgCl_2_, 0.25 mM dNTP mix, 0.125 μl RNasin, 0.25 μl random primers, and 0.175 μl AMVRT. A 1:4 dilution of the RT reaction was used as described above. All experiments were performed with at least three biological replicates with three technical replicates each.

For qualitative reverse transcription PCR (RT-PCR) analyses, a dilution of 1:125 of cDNA was used in a PCR using the GoTaq Green Master Mix with 0.2 μM of the following primers: cctgcccagttggtttagc, forward primer; gctgccaaattaatacggaag, reverse primer. The initial denaturation step was performed at 95°C for 2 min. For a total of 33 cycles, denaturation was performed at 95°C, followed by annealing at 51°C, and elongation at 72°C for 30 s each. The final elongation was performed at 72°C for 2 min. A control was performed for ribosomal RNA as previously detailed (Pattanaik and Montgomery, 2010).

### Statistical Analyses

All experiments were performed with at least three independent biological replicates and results are presented as the mean (±SD), except for analyses of cellular morphology where mean (±SE) was calculated. Three technical replicates were analyzed from each of three biological replicates in qPCR experiments. Data were analyzed with a two-tailed, unpaired Student’s *t*-test. If more than two sample groups were analyzed, a one-way ANOVA test for independent samples was performed. If a significant F ratio was obtained in the ANOVA analyses, a Tukey HSD test was performed.

## Results

### *FdTSPO* is Homologous to Known TSPO Proteins and is Co-localized with Photosynthesis-associated Genes

**FIGURE 1:**
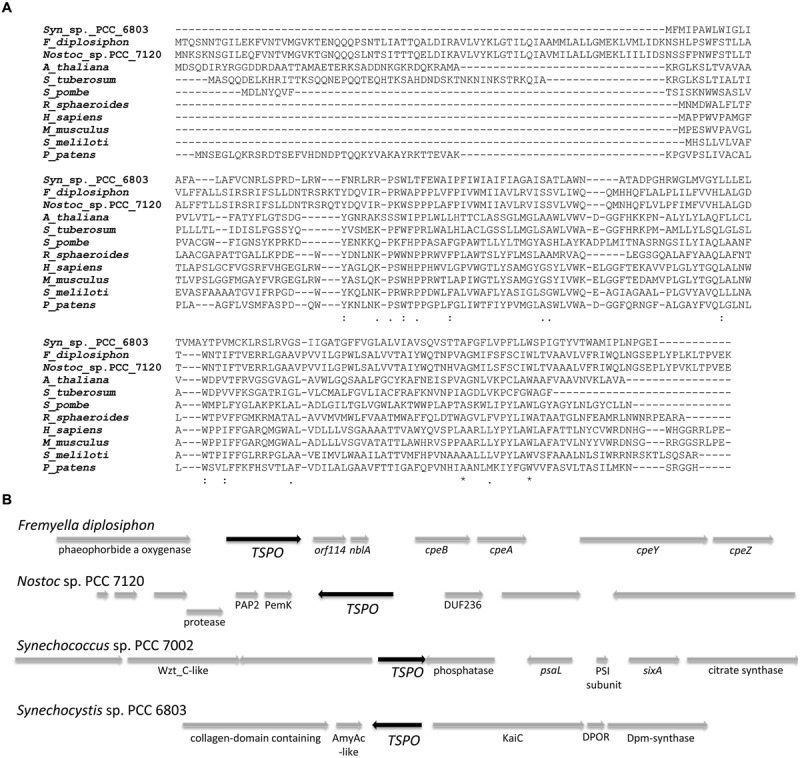
**Sequence alignment and representation of genomic context of the putative tryptophan-rich sensor protein (*TSPO*) in *Fremyella diplosiphon* compared to other cyanobacterial genomes. (A)** Sequence alignment of TSPO homologs from *Synechocystis* sp. PCC 6803 (*Syn*_sp._PCC_6803; Accession: BAA18606.1), *F. diplosiphon* (*F_diplosiphon*; Accession: AAT36314.1), *Nostoc* sp. PCC 7120 (*Nostoc*_sp._PCC_7120; Accession: WP_010997158.1), *Arabidopsis thaliana* (*A_thaliana* Accession: NP_566110), *Solanum tuberosum* (*S_tuberosum*; Accession: CAH10765.1), *Schizosaccharomyces pombe* (*S_pombe*; Accession: NP_595490.2), *Rhodobacter sphaeroides* 2.4.1 (*R_sphaeroides*; Accession: WP_002720433.1), *Homo sapiens* (*H_sapiens*; Accession: AAA03652.1), *Mus musculus* (*M_musculus*; Accession: NP_033905.3), *Sinorhizobium meliloti* (*S_meliloti*; Accession: WP_012881280.1), and *Physcomitrella patens* (*P_patens*; Accession: ABG37902.1) was generated with CLUSTALW by MUSCLE (3.8) at http://www.ebi.ac.uk/Tools/msa/muscle/ (Edgar, 2004). Symbols below alignment: asterisk indicates positions which have a single, fully conserved residue; colon indicates conservation between groups of strongly similar properties – scoring > 0.5 in the Gonnet PAM 250 matrix; period indicates conservation between groups of weakly similar properties – scoring ≤ 0.5 in the Gonnet PAM 250 matrix. **(B)** Arrows represent open reading frames with a black arrow for *TSPO*. Annotated or known genes have names below the arrows, whereas hypothetical or unknown genes are not labeled.

An alignment of *Fd*TSPO with TSPO homologs from mammals, fungi, plants and bacteria shows the presence of an N-terminal extension in the *Fd*TSPO sequence (**Figure 1A**). A similar extension functions as a signaling sequence in *Arabidopsis*, where alternative start codons also have been associated with different subcellular localization (Balsemão-Pires et al., 2011). As the prokaryotic *F. diplosiphon* does not have the different subcellular organelles associated with *At*TSPO targeting in *Arabidopsis*, the function of this extension in *Fd*TSPO is likely distinct. A possible alternative start codon is located at position M70 in *Fd*TSPO. Notably, putative TSPO homologs of more closely related cyanobacteria like *Nostoc* show a similar size to that of *Fd*TSPO, which is distinct from the predicted length of a TSPO homolog from *Synechocystis* (**Figure 1A**).

Fd*TSPO* is located in the *F. diplosiphon* genome with unknown gene *ORF114, nblA1* (Baier et al., 2004), *cpeBA* genes (Mazel et al., 1986), and *cpeYZ* genes (Tandeau de Marsac et al., 1988) downstream (**Figure 1B**). Upstream of *FdTSPO* is an ORF with sequence homology to a gene encoding pheophorbide *a* oxygenase, an enzyme that is involved in chlorophyll breakdown (Hörtensteiner et al., 1995). In *F. diplosiphon*, the *cpeBA* genes encode subunits of GL-absorbing photosynthetic pigment PE and the *cpeYZ* operon encodes the respective lyases that attach the phycoerythrobilin chromophore to the PE subunit (Kahn et al., 1997; Biswas et al., 2011). The expression of these genes is up-regulated under GL (Mazel et al., 1986; Kahn et al., 1997), as has been previously reported for *FdTSPO* (Stowe-Evans et al., 2004). Two classes of *nblA* genes are distinguished, *nblA1* and *nblA2.* NblA1/2 dimers function in the regulated degradation of PBSs upon nitrogen starvation by acting as an adapter that mediates protease digestion of the PBS in cyanobacteria (Collier and Grossman, 1994; Karradt et al., 2008; Baier et al., 2014). The *nblA1* gene is transcriptionally regulated by nitrogen availability in *F. diplosiphon* (Luque et al., 2003). The clustering of *FdTSPO* with genes associated with photosynthesis, specifically with biosynthesis and degradation of tetrapyrroles or tetrapyrrole-containing complexes, suggests a physiologically relevant connection of *TSPO* with these processes.

Based on the genomic context of *TSPO* in *F. diplosiphon*, we analyzed the genetic context of other cyanobacterial *TSPO-*like genes using the Gene Context Tool (Martinez-Guerrero et al., 2008). Although not a direct parallel to the gene context found in *F. diplosiphon*, photosynthesis-related genes are present in the vicinity of *TSPO* in *Synechococcus* sp. PCC 7002 [photosystem I subunit and *psaL*, which is required for PSI trimer formation in cyanobacteria (Chitnis and Chitnis, 1993)] and in *Synechocystis* sp. PCC 6803 [protochlorophyllide reductase, DPOR, which is involved in light-independent chlorophyll synthesis (Armstrong, 1998), (**Figure 1B**)]. Annotated entries for *TSPO*-like cyanobacterial genes are limited and a homolog is not found in all sequenced cyanobacterial genomes and/or identification is limited by lack of annotation of *TSPO*-homologs in known genomes. Therefore, the possibility that the same or a similar genetic context as that observed for *F. diplosiphon* is present in other cyanobacterial genomes cannot be fully excluded. The current analysis highlights the diversity of *TSPO*-flanking sequences in different cyanobacterial species.

### *FdTSPO* is Up-regulated under Green Light

**FIGURE 2:**
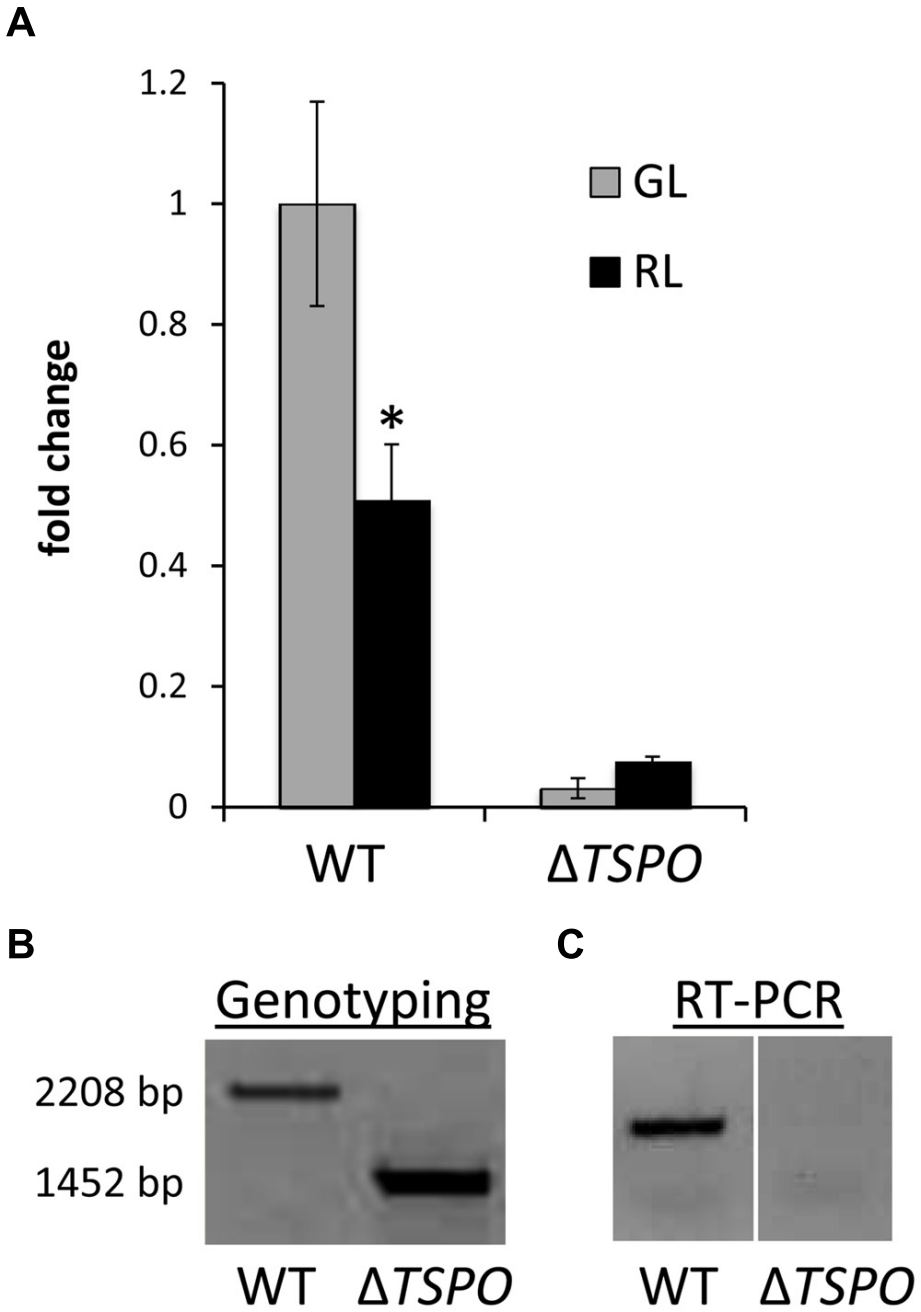
**Genotype and reverse transcription analyses of *FdTSPO* in *F. diplosiphon* SF33 wild-type and Δ*FdTSPO* mutant strains. (A)** Quantitative real-time reverse transcription PCR (qRT-PCR) analysis for wild type (WT) and Δ*FdTSPO* mutant strains grown at 10 μmol m^-2^ s^-1^ in either green light (GL) or red light (RL). Relative expression level compared to *ORF10B* gene is shown (±SD, *n* = 3). Fold change of expression of *FdTSPO* is shown relative to WT cells grown in GL. The asterisk indicates a significant difference (^∗^*p* = 0.011). *P*-values were determined using an unpaired, two-tailed Student’s *t*-test comparing WT in GL vs. RL. **(B)** PCR analysis of *FdTSPO*-containing genomic region in WT and Δ*FdTSPO* strains. **(C)** Qualitative reverse transcription analysis of *FdTSPO* expression in WT and Δ*FdTSPO* background.

As noted above, the upregulation of *FdTSPO* expression under GL conditions has been previously reported for microarray-based analyses (Stowe-Evans et al., 2004). We assessed levels of *FdTSPO* mRNA based on RNAseq analysis of *F. diplosiphon* cells grown under GL and RL (Pattanaik et al., 2014). *FdTSPO* levels were ∼2.5-fold higher under GL compared to RL in WT (**Table 1**). The higher accumulation in WT in GL compared to RL was confirmed by quantitative real-time, reverse transcription PCR (qRT-PCR; **Figure 2A**). *FdTSPO* transcript levels were higher in a mutant lacking functional RcaE photoreceptor (i.e., Δ*rcaE* strain) that controls the CCA response, but levels were still higher on average in GL compared to RL for this Δ*rcaE* strain, i.e., 1.7-fold higher in GL (**Table 1**). A similar observation was made under iron deficiency for WT, with significantly higher levels of *FdTSPO* mRNA in GL than RL. However, no light quality-dependent regulation of *FdTSPO* levels was observed under iron depletion in Δ*rcaE* (i.e., Δ*rcaE*/-Fe) with overall lower levels of *FdTSPO* mRNA than for any other condition or strain apart from WT cells under RL (**Table 1**). Our lab previously reported an involvement of RcaE in regulation of iron acclimation in *F. diplosiphon* (Pattanaik et al., 2014). Additionally, an association of TSPO and iron has been suggested for *Pseudomonas* (Leneveu-Jenvrin et al., 2014). Our current data show that *TSPO* expression is regulated by light quality and possibly by iron content, although no significant difference in internal iron availability was observed between WT and Δ*FdTSPO* mutant strains (Supplementary Figure S1).

**Table 1:** RNA sequencing-based analyses of *FdTSPO* accumulation in *F. diplosiphon* SF33 wild type (WT) and RcaE photoreceptor mutant (Δ*rcaE*) under green light (GL) or red light (RL) growth in replete (+Fe) or iron-limited (-Fe) medium.

Sample	No. of reads/WT		Fold change^a^	*p*-value^b^		
	GL	RL	GL/RL	GL vs. RL	WT vs. sample	
					GL	RL
WT	16.8	6.65	2.5	0.03	–	–
Δ*rcaE*	25.6	14.9	1.7	0.19	0.35	0.05
WT/-Fe	27.9	10.8	2.6	0.03	0.14	0.17
Δ*rcaE*/-Fe	8.7	9.0	0.97	0.88	0.37	0.44

*The ORF representing *FdTSPO* was determined by comparison to *Anabaena variabilis* ATCC29413 annotated proteins using BLASTX and a cut-off *e*-value of 0.0001.*

*^a^Fold change: differential expression analysis between two light treatments was carried out for each strain. Data were extracted for RNA-seq data described in Pattanaik et al. (2014).*

*^b^*P*-value: the significance value was calculated for counts for indicated comparison using unpaired two-tailed Student’s *t*-test.*

### *TSPO* is Not Essential in *F. diplosiphon*

A *F. diplosiphon* mutant lacking *FdTSPO*, deleting which was designated Δ*FdTSPO*, was generated by the locus for *FdTSPO* via homologous recombination (Cobley et al., 1993; Pattanaik and Montgomery, 2010). Genotyping with primers located just outside the *FdTSPO* gene did not result in a signal for WT copies (i.e., 2208 bp product) in the mutant (**Figure 2B**). Furthermore, a lack of *FdTSPO* transcript accumulation was confirmed by qualitative reverse transcription PCR (**Figure 2C**) and qRT-PCR (**Figure 2A**). As the Δ*FdTSPO* mutant was able to grow in the absence of WT copies of the gene, *Fd*TSPO does not appear essential for the cell, which is consistent with reports from eukaryotic and bacterial TSPO studies (Yeliseev and Kaplan, 1995; Guillaumot et al., 2009; Tu et al., 2014).

### Δ*FdTSPO* Pigmentation and Growth

**FIGURE 3:**
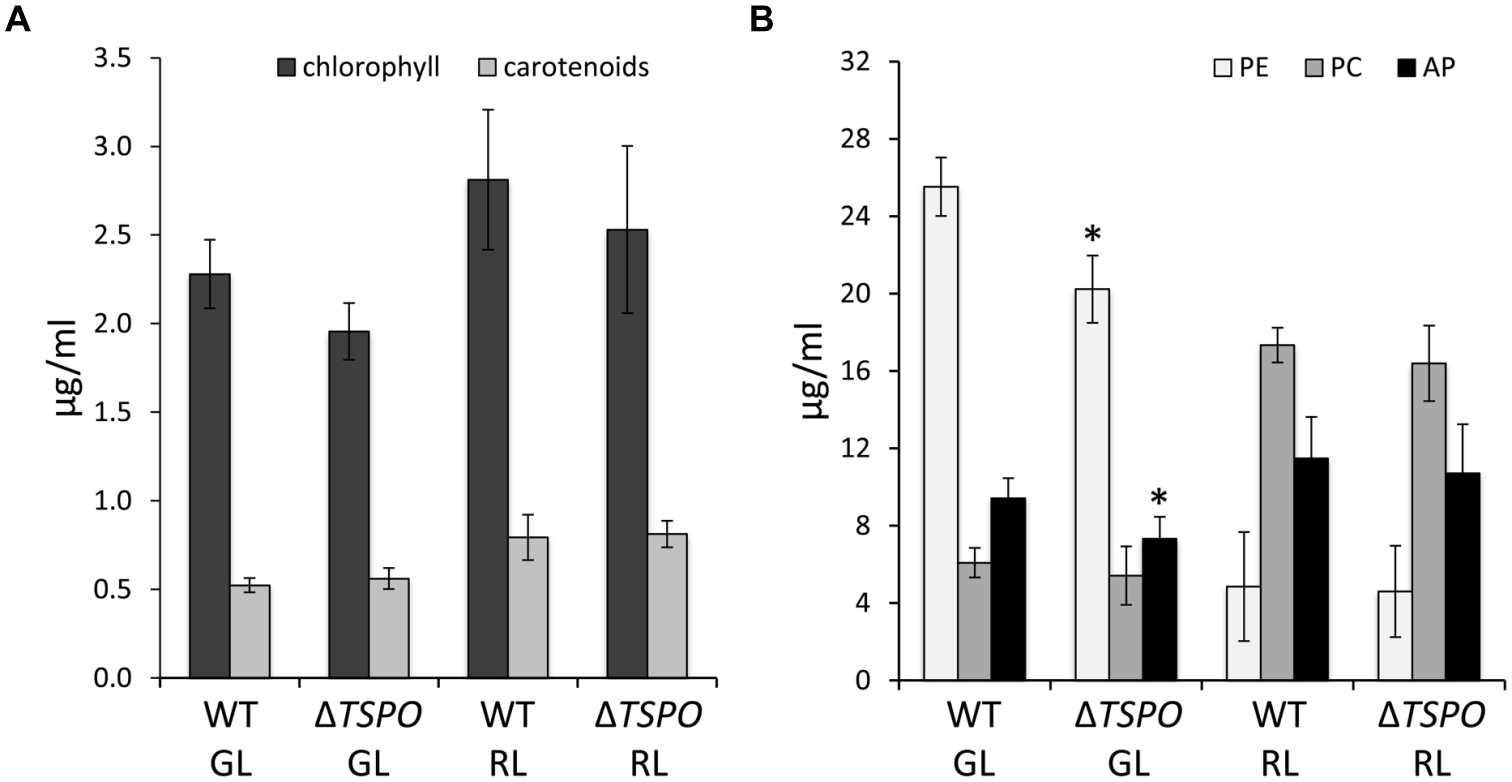
**Pigment content of *F. diplosiphon* SF33 wild-type and Δ*FdTSPO* mutant strains under standard conditions.** WT and *FdTSPO* mutant (Δ*TSPO*) cells were grown at ∼10 μmol m^-2^ s^-1^ under GL or RL. **(A)** Chlorophyll and carotenoid contents and **(B)** phycobiliprotein levels were measured from an equal aliquot of cells as determined by optical density at 750 nm (OD_750_). Bars represent mean (±SD, *n* ≥ 6). Asterisks indicate a significant difference (^∗^*p* < 0.01) between WT and mutant for the same light condition as determined by ANOVA and Tukey test.

Whole cell absorption spectra of Δ*FdTSPO* mutant cells did not differ from the WT under standard light conditions of ∼10 μmol m^-2^ s^-1^ in GL or RL (Supplementary Figure S2). We analyzed the abundance of the phycobiliproteins PE, PC and allophycocyanin (AP), and the concentrations of chlorophyll and carotenoids in GL- and RL-grown WT and mutant cells (**Figure 3**). Levels of chlorophyll and carotenoids were not significantly different in the mutant compared to the WT. Phycobiliprotein content was slightly lower in the mutant compared to the WT only for PE and AP levels under GL, with a maximum difference of a 20.7% reduction of PE levels in the mutant under GL compared to the WT (**Figure 3**). Notably, the defect in PE and AP in GL corresponds to the light conditions under which *FdTSPO* is upregulated in WT. No significant differences in growth of the mutant compared to the WT under standard conditions at ∼10 μmol m^-2^ s^-1^ were observed (Supplementary Figure S3).

### A Lack of *FdTSPO* is Correlated with Responses to Stress

**FIGURE 4:**
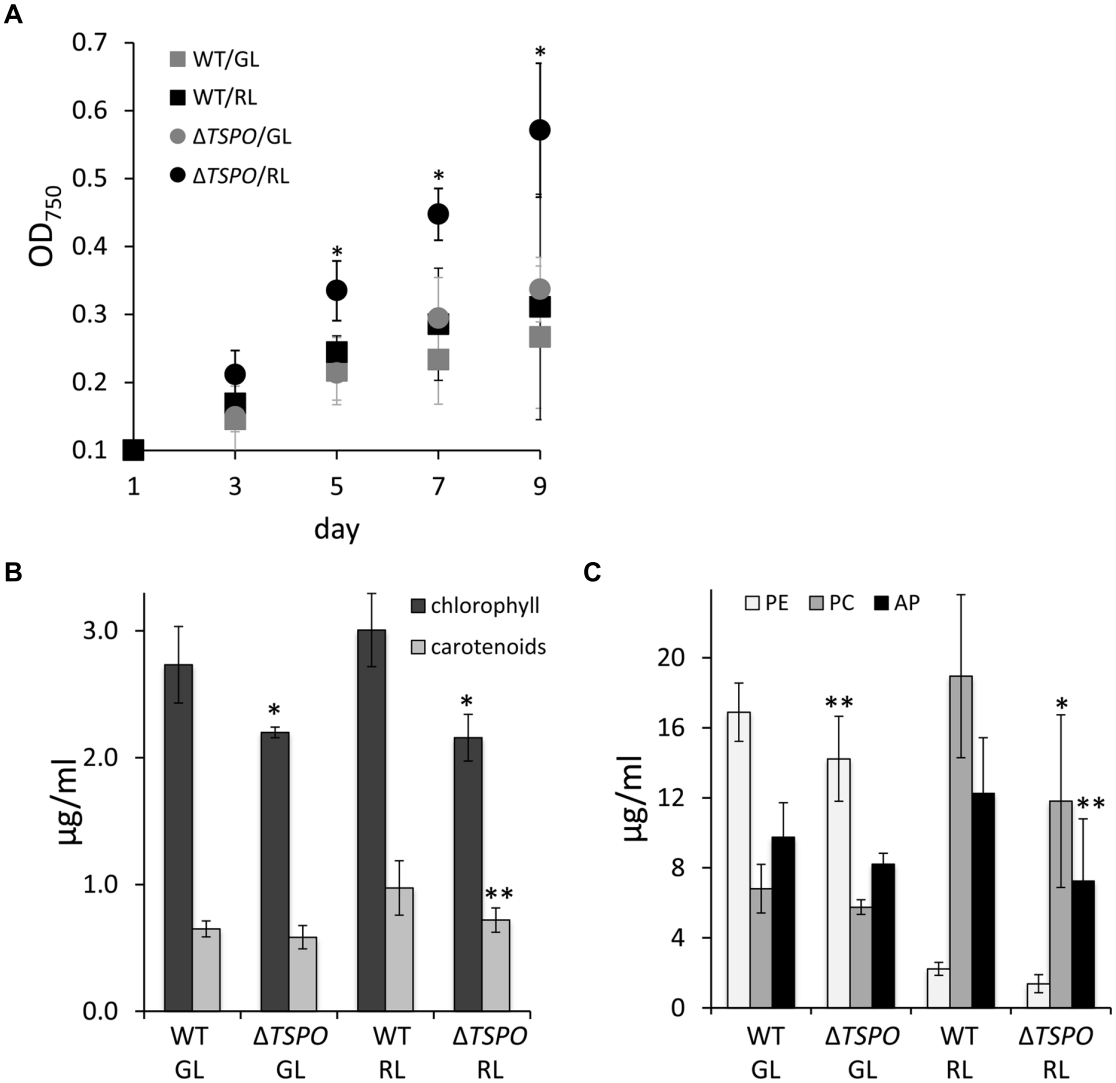
**Growth and pigment content in *F. diplosiphon* SF33 wild-type and Δ*FdTSPO* mutant strains under salt stress. (A)** WT and *FdTSPO* mutant (Δ*TSPO*) cells were grown at ∼10 μmol m^-2^ s^-1^ in BG-11/HEPES medium containing 200 mM NaCl (w/v) and growth was monitored over 8 days at OD_750_. **(B)** Chlorophyll and carotenoid contents and **(C)** phycobiliprotein levels were determined after growth in BG-11/HEPES medium containing 200 mM NaCl at ∼10 μmol m^-2^ s^-1^ of GL or RL. Bars represent mean (±SD, *n* ≥ 6). Asterisks indicate a significant difference (^∗^*p* < 0.01, ^∗∗^*p* < 0.05) between WT and mutant for the same light condition **(A,B)** or at a given time point **(A)**, as determined by ANOVA and Tukey test.

A growth-deficient phenotype due to salt stress was previously reported in WT *F. diplosiphon*, with the strongest negative impact on growth being observed at 200 mM NaCl (Singh and Montgomery, 2013b). The Δ*FdTSPO* mutant performed significantly better than the WT under salt stress in RL in terms of growth (**Figure 4A**). Notably, overall photosynthetic pigment levels were lower in the mutant compared to the WT during salt stress (**Figures 4B,C**). Chlorophyll levels were significantly lower in both light conditions in the mutant, whereas carotenoid levels were significantly lower in the mutant under RL compared to the WT (**Figure 4B**). Although not significant in the case of PE, a reduction of ∼40% in PC, PE, and AP content was observed under RL (**Figure 4C**), conditions for which differences in growth were greatest (**Figure 4A**). This salt-induced increase in growth for Δ*FdTSPO* relative to WT was specific to TSPO function as it was lost when the Δ*FdTSPO* mutant was complemented with a WT *TSPO* gene driven by its own promoter (Supplementary Figure S4).

**FIGURE 5:**
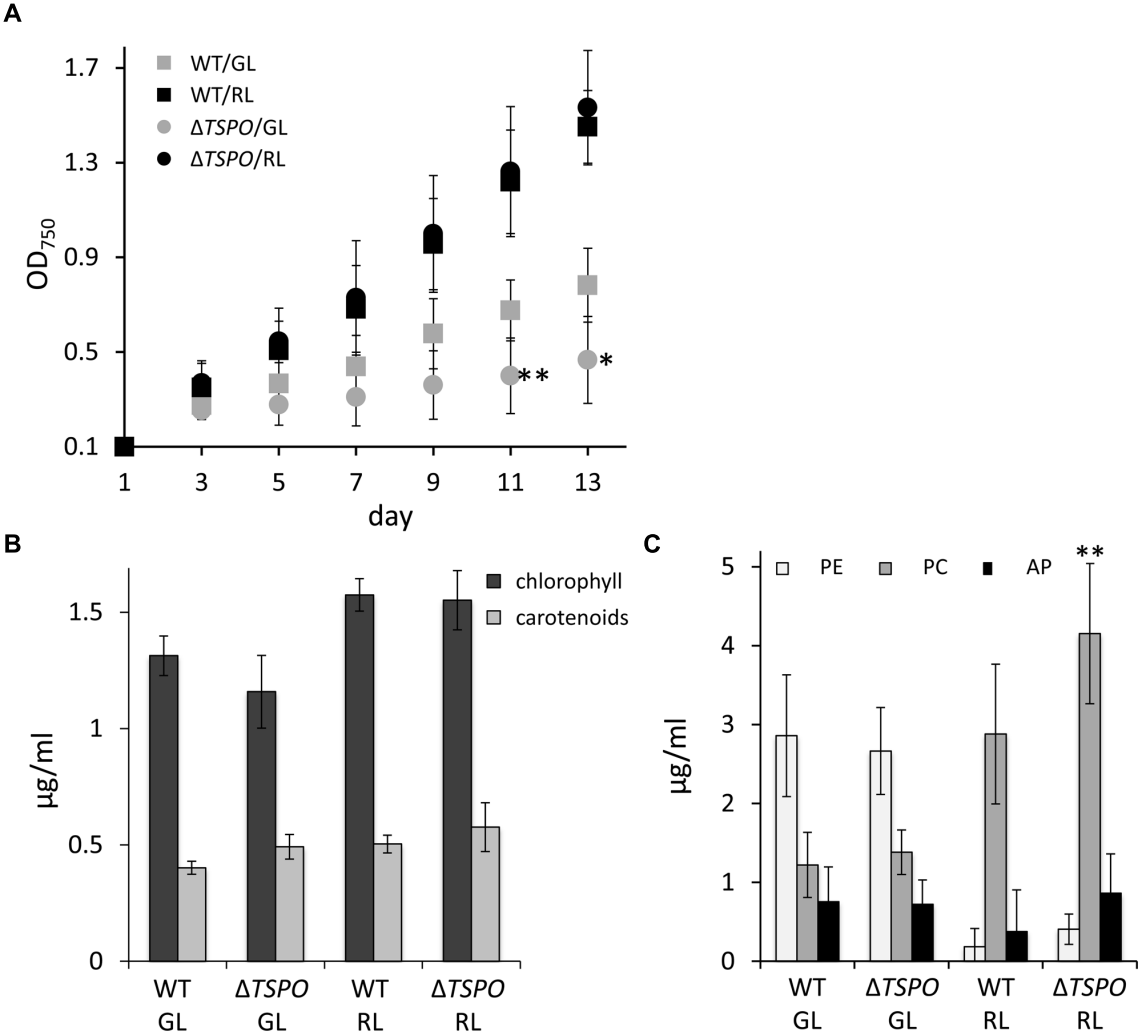
**Growth and pigment content in *F. diplosiphon* SF33 wild-type and Δ*FdTSPO* mutant strains under osmotic stress. (A)** WT and *FdTSPO* mutant (Δ*TSPO*) cells were grown at ∼10 μmol m^-2^ s^-1^ in BG-11/HEPES medium containing 400 mM sorbitol (w/v) and growth was monitored over 12 days at OD_750_. **(B)** Chlorophyll and carotenoid contents and **(C)** phycobiliprotein levels were determined after growth in BG-11/HEPES medium containing 400 mM sorbitol at ∼10 μmol m^-2^ s^-1^ of GL or RL. Bars represent mean (±SD, *n* = 6). Asterisks indicate a significant difference (^∗^*p* < 0.01,^∗∗^*p* < 0.05) between WT and mutant for the same light condition **(A,B)** or at a given time point **(A)**, as determined by ANOVA and Tukey test.

Salt stress can induce ionic stress, as well as lower the water potential of cells, thereby resulting in osmotic stress (Hagemann, 2011). We, thus, tested whether part of the alterations in growth observed in response to salt treatment, could also be observed in response to osmotic stress. Osmotic stress induced by sorbitol treatment resulted in distinct responses for the Δ*FdTSPO* mutant compared to WT. We observed impairment in growth for the Δ*FdTSPO* mutant compared to WT under GL (**Figure 5A**). Overall the negative effect of sorbitol on growth was much greater in GL than in RL for WT and mutant. Chlorophyll and carotenoid levels were not impacted by sorbitol treatment (**Figure 5B**). However, significantly higher accumulation of PC was observed for the Δ*FdTSPO* mutant under RL in sorbitol-treated cells (**Figure 5C**). No other significant impacts on the accumulation of phycobiliproteins were noted in either GL or RL in response to sorbitol (**Figure 5C**).

### Δ*FdTSPO* is More Sensitive to Methyl Viologen-induced Oxidative Stress but Shows Less ROS Formation under Salt Stress Compared to WT

**FIGURE 6:**
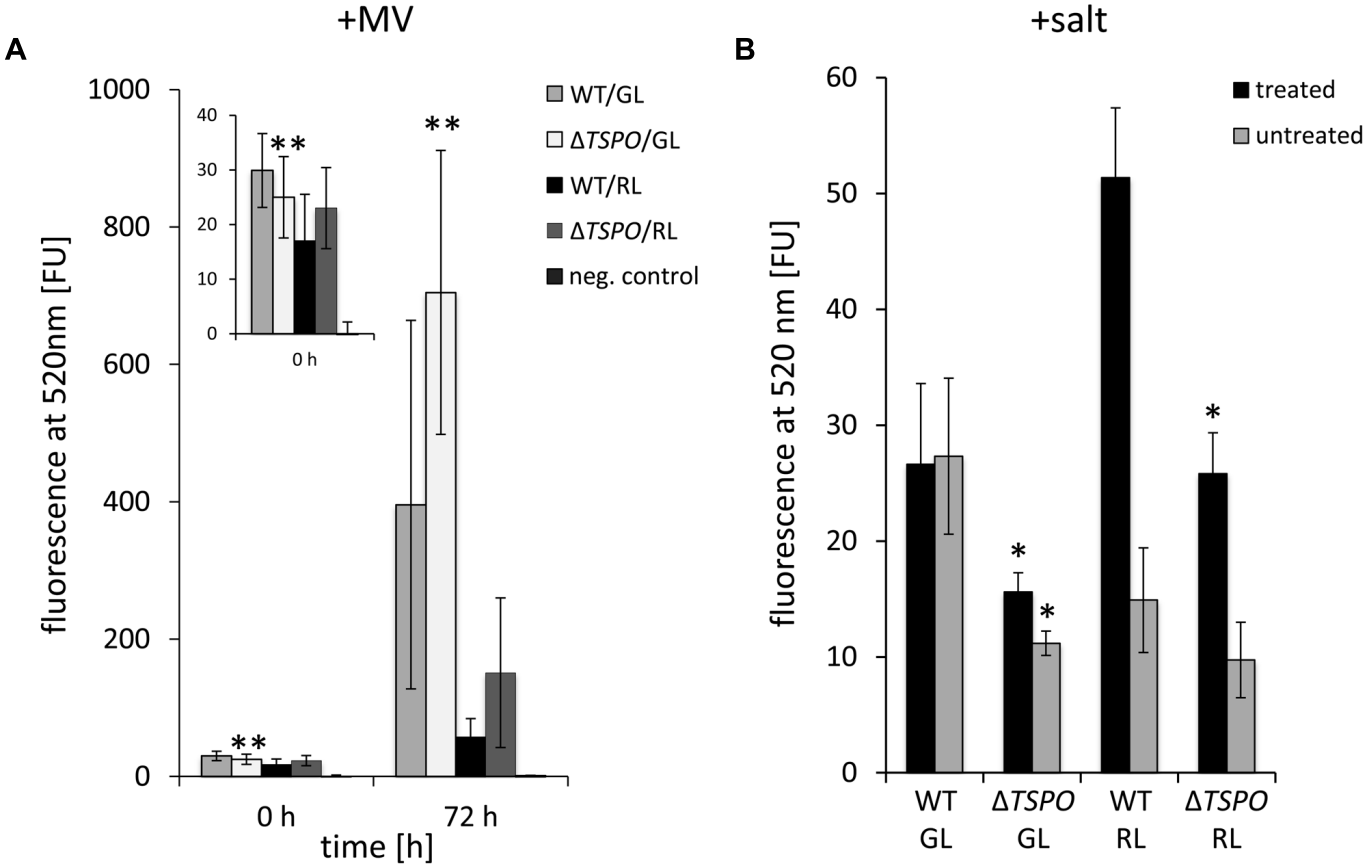
**Reactive oxygen species (ROS) content in *F. diplosiphon* SF33 wild-type and Δ*FdTSPO* mutant strains treated with salt and methyl viologen (MV). (A)** ROS was measured by DCF-dependent fluorescence at 520 nm before treatment (0 h) and 3 days after addition of 0.3 μM MV (72 h) for WT and *FdTSPO* mutant (Δ*TSPO*) cells that were adapted to either GL or RL at ∼ 10 μmol m^-2^ s^-1^. **(B)** ROS was measured by DCF-dependent fluorescence at 520 nm after 6 days of treatment with 200 mM NaCl (w/v; black bars) or untreated control (gray bars). Bars represent mean (±SD; *n* ≥ 3). Asterisks indicate a significant difference (^∗^*p* < 0.01, ^∗∗^*p* < 0.05) between WT and mutant for the same light condition at a given time point, as determined by ANOVA and Tukey test.

A potential association of *Fd*TSPO with ROS levels was investigated as elevated oxidative stress has been correlated with slower growth and/or cell death in photosynthetic organisms (Busch and Montgomery, 2015). Furthermore, salt stress, which is associated with TSPO function in *F. diplosiphon*, can cause oxidative stress in cyanobacteria (Srivastava et al., 2008; Srivastava, 2010). TSPO has also been implicated in stress responses in plants (Frank et al., 2007; Guillaumot et al., 2009) and animals (Papadopoulos et al., 2006). *AtTSPO* overexpression was associated with increased ROS levels and sensitivity to salt in *Arabidopsis*, which suggested that TSPO enhances oxidative stress signaling in this organism (Vanhee et al., 2011). A *PpTSPO1* deletion mutant in the moss *Physcomitrella patens* exhibited higher ROS formation under salt stress (Frank et al., 2007). In a later study, the Δ*PpTSPO1* mutant was shown to have increased levels of superoxide upon treatment with a fungal elicitor (Lehtonen et al., 2012). We, therefore, tested the impact of induced oxidative stress on WT and Δ*FdTSPO* cells. We analyzed ROS levels with the ROS-sensitive dichlorodihydrofluorescein diacetate (DCFH-DA) in GL- and RL-adapted Δ*FdTSPO* mutant and WT cells and in similarly grown cells after treatment with the electron acceptor MV or salt (**Figure 6**).

Green light- and RL-adapted cells showed only minor differences in ROS content between WT and mutant under standard conditions, with the mutant exhibiting significantly lower ROS levels under GL (**Figure 6A**). After 72 h of MV treatment, GL-grown Δ*FdTSPO* mutant cells exhibited a significantly higher ROS content than WT (**Figure 6A**). This result implies that the mutant is less equipped to cope with externally applied MV-induced stress. In the same manner, ROS content was measured in salt-stressed cells. The mutant exhibited significantly lower salt-induced ROS-levels after 72 h of treatment with 200 mM NaCl than WT under RL (**Figure 6B**). Notably, salt treatment did not result in an increase in ROS levels in WT under GL.

### *FdTSPO* is Transiently Up-regulated Under Stress

**FIGURE 7:**
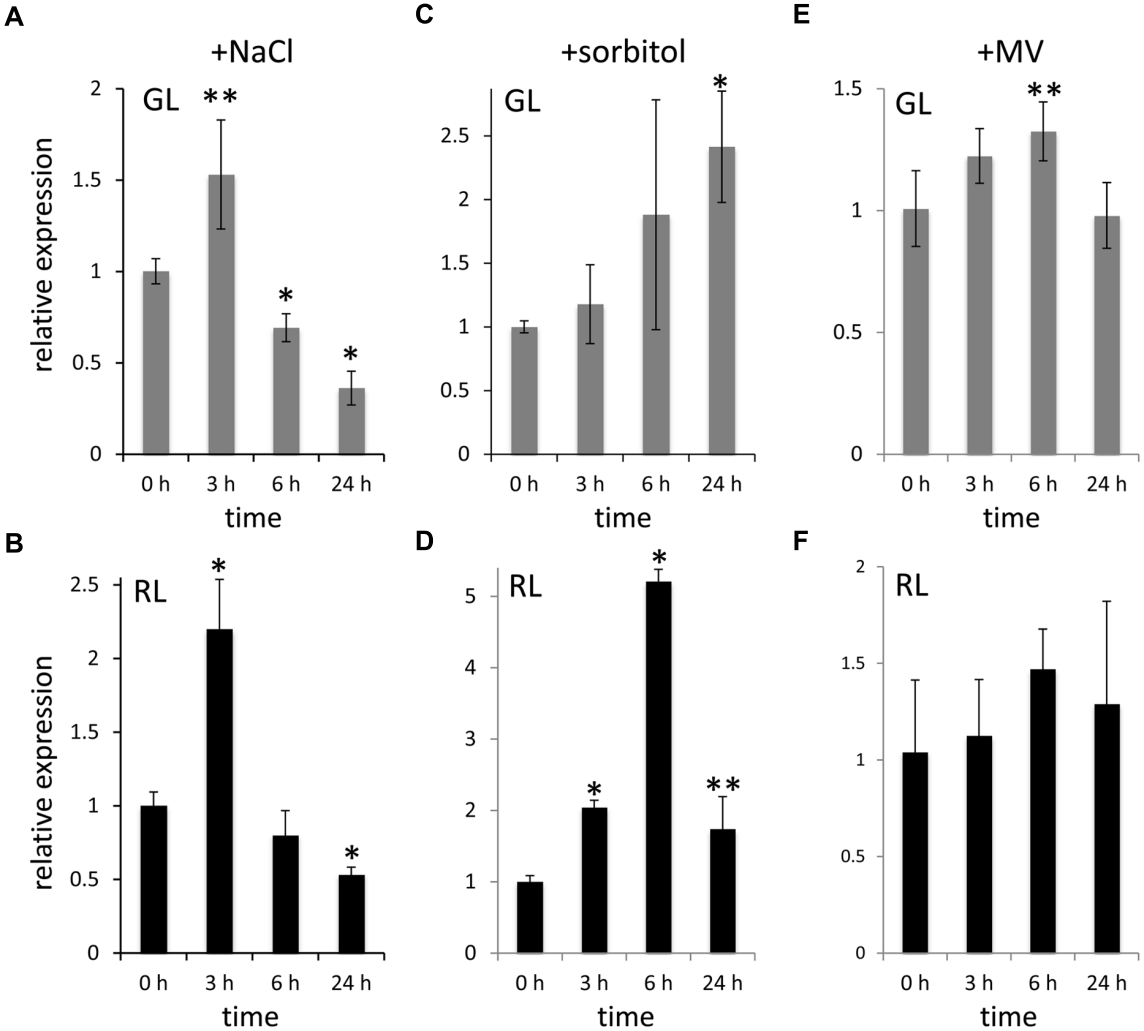
**Stress-dependent upregulation of *FdTSPO* transcript levels.** Samples for quantitative real-time reverse transcription PCR (qRT-PCR) were taken before (0 h) and after (3 h, 6 h, or 24 h) addition of **(A,B)** 200 mM NaCl (w/v), **(C,D)** 400 mM sorbitol (w/v) or **(E,F)** 0.3 μM methyl viologen (MV) to WT cells in GL and RL to assess *FdTSPO* transcript accumulation in response to stress. Relative expression level compared to *ORF10B* gene is shown (±SD, *n* = 3). Expression of *FdTSPO* is shown relative to expression levels in WT cells at time 0 h in the respective light condition. *P*-values determined using unpaired, two-tailed Student’s *t*-test comparing expression levels before treatment with levels after salt, sorbitol, or MV treatment in either GL or RL (^∗^*p* < 0.01, ^∗∗^*p* < 0.05).

The altered response of the Δ*FdTSPO* mutant to salt treatment and osmotic shock suggests a role for *Fd*TSPO in responses to stress in *F. diplosiphon*. To further understand the relationship between *Fd*TSPO and salt stress, osmotic stress, and induced oxidative stress, we followed *FdTSPO* transcript accumulation over a period of 1 day, i.e., 3, 6, and 24 h after addition of 200 mM salt, treatment with osmotic stress inducer sorbitol at 400 mM, or 0.3 μM of oxidative stress inducer MV (**Figure 7**). After exhibiting a transient two-fold increase under RL and ∼1.5-fold increase under GL at 3 h, *FdTSPO* levels fell below the values observed before addition of salt (i.e., at 0 h) after 6 and 24 h of stress (**Figures 7A,B**). The transient upregulation of *FdTSPO* implies a function in the early stages of salt stress response. *FdTSPO* was also transiently upregulated by ∼fivefold at 6 h under RL with sorbitol treatment (**Figure 7C**). Expression was highest at ∼2.5-fold after 24 h of sorbitol treatment in GL (**Figure 7D**). By comparison, after treatment with MV only a modest increase in expression was observed. After 6 h in GL, *FdTSPO* expression was significantly increased by ∼1.3, with no significant upregulation of expression noted under RL (**Figures 7E,F**). Taken together, *FdTSPO* exhibited higher upregulation under stress conditions that were likely to occur under physiological conditions, i.e., salt and osmotic stresses.

### Δ*FdTSPO* Mutant Exhibits Changed Morphology under Different Light Conditions

**FIGURE 8:**
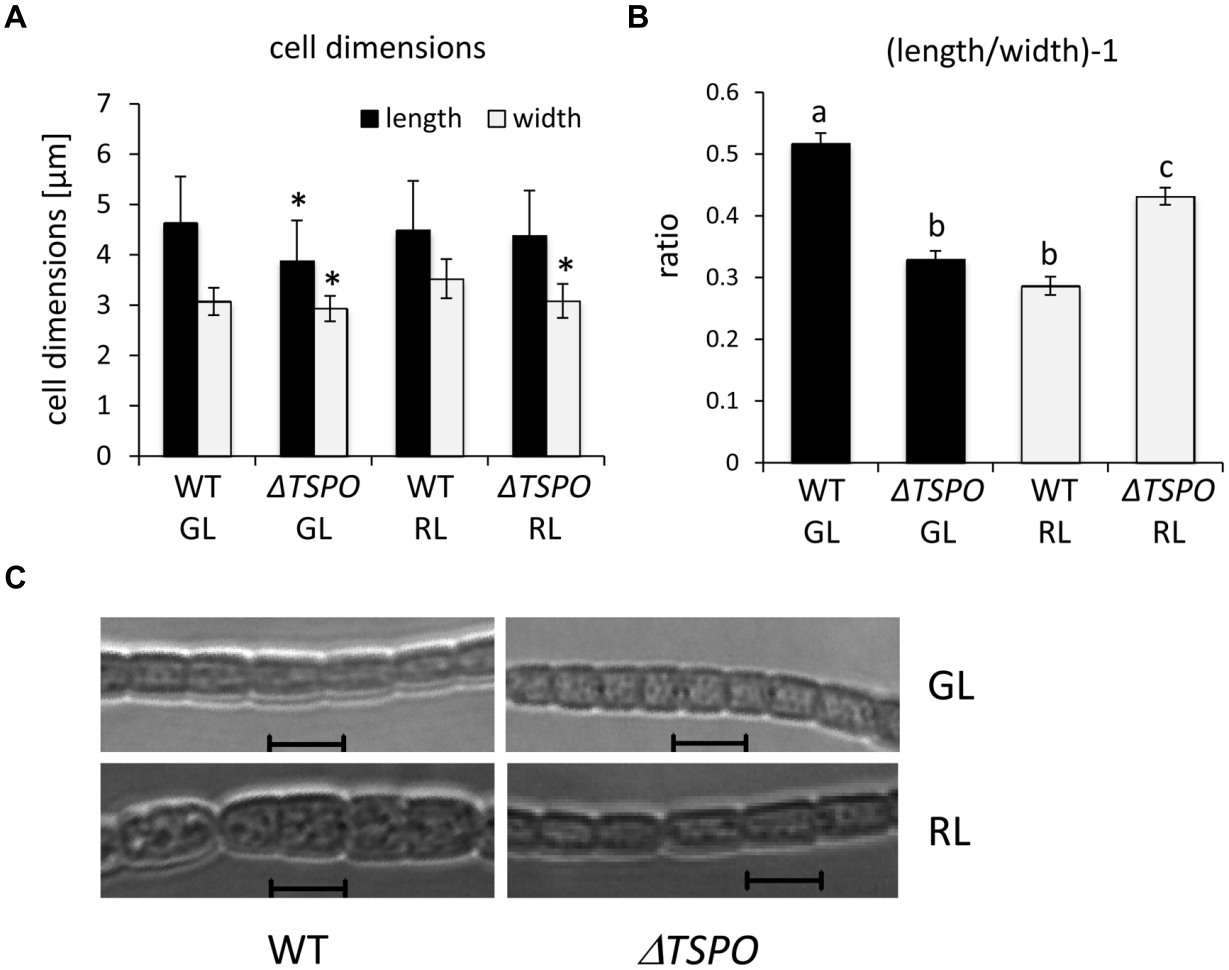
**Analyses of the cellular morphology of WT and Δ*FdTSPO* strains of *F. diplosiphon* grown under GL or RL. (A,B)** Cell length and width measurements of WT or mutant (Δ*TSPO*) cells were obtained for cultures grown in GL or RL at ∼10 μmol m^-2^ s^-1^. These measurements were used to calculate the length-to-width ratio, i.e., (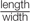) - 1. Bars represent mean (±SE) for a total of at least 370 individual cells from four independent biological replicates. Asterisks indicate a significant difference (^∗^*p* < 0.01) for the mutant relative to WT for the same light condition and similar letters indicate a homogenous mean group and different letters indicate a significant difference (^∗^*p* < 0.01), as determined by ANOVA and Tukey test. **(C)** Representative optical slices from a *Z*-series of differential interference contrast (DIC) images of WT or mutant cells grown under GL or RL for 72 h. Images were acquired using a 40× oil immersion objective with 2× zoom setting. Bars, 5 μm.

Given differential regulation of *FdTSPO* mRNA levels under GL vs. RL, we assessed additional phenotypes known to be GL- and RL-responsive in *F. diplosiphon*. WT cells are more round under RL, whereas cells are more rectangular under GL (Bennett and Bogorad, 1973; Bordowitz and Montgomery, 2008). Δ*FdTSPO* cells are smaller under GL and RL (**Figure 8A**) and show a lower length:width ratio than the WT under GL, whereas the ratio is higher for the mutant than WT under RL (**Figure 8B**). Based on the observed difference in the length:width ratio, the Δ*FdTSPO* mutant seems to exhibit a morphology phenotype that is the inverse of that of the WT. Δ*FdTSPO* mutant cells indeed appear more round in GL and more rectangular under RL (**Figure 8C**). This effect could be due to *Fd*TSPO directly impacting the regulation of cell morphology. Nevertheless, we cannot exclude the possibility that the altered cell morphology of the Δ*FdTSPO* mutant is due to some indirect effect caused by the lack of *Fd*TSPO protein, which is a membrane protein.

## Discussion

Tryptophan-rich sensory protein is found in many organisms and despite many efforts the complete function of this protein is still not fully elucidated (Batoko et al., 2015; Campanella, 2015; Gut et al., 2015; Kim and Yu, 2015; Selvaraj and Stocco, 2015). We have analyzed TSPO from the chromatically acclimating cyanobacterium *F. diplosiphon* to better understand its function in photosynthetic organisms and to elucidate its function in cyanobacteria, for which there have been no published reports of functional studies. The level of sequence identity between some cyanobacterial TSPO homologs and the distinct genomic contexts observed in different organisms (**Figure 1**) may be explained by varying organismal habitats and different types of chromatic acclimation found in the cyanobacteria phylum (Carr and Whitton, 1982; Whitton and Potts, 2000). Functions unique to TSPO in a type III chromatically adapting cyanobacterium such as *F. diplosiphon*, which exhibits light-dependent changes in phycobiliprotein content (Bennett and Bogorad, 1973) and thus distinct light-dependent tetrapyrrole demands, are feasible.

The deletion of *FdTSPO* from the genome did not result in a great impact on cellular growth or pigment content under standard growth conditions (**Figure 3**, Supplementary Figures S2 and S3). By comparison, TSPO negatively affects photosynthetic pigment accumulation in *Rhodobacter sphaeroides* through negative regulation of transcription of carotenoid and bacteriochlorophyll biosynthesis genes, i.e., aerobically grown Δ*RsTSPO* mutant cells have higher levels of carotenoids and bacteriochlorophyll than WT; whereas TSPO overexpression results in reduced pigment content compared to the WT (Yeliseev and Kaplan, 1995). Also, overexpression of *AtTSPO* resulted in lower chlorophyll levels compared to the WT in *Arabidopsis* cells exposed to light (Guillaumot et al., 2009). Thus, *Fd*TSPO might differ in its function from other photosynthetic organisms or a lack of *Fd*TSPO may be compensated for by an unknown mechanism in *F. diplosiphon* resulting in only relatively small differences in the levels of photosynthetic pigmentation in the Δ*FdTSPO* mutant strain. Although Δ*RsTSPO* mutant cells were observed to accumulate higher pigment levels than WT in aerobic conditions, pigment levels converged to similar levels in the distinct strains after an acclimation period when cells were switched from aerobic to semi-aerobic growth (Yeliseev and Kaplan, 1995). Thus, the role for *Rs*TSPO appears fine-tuned for regulating growth of this organism that exhibits anaerobic phototrophic growth and aerobic heterotrophic growth, very distinct from the aerobic phototroph *F. diplosiphon*. However, this role of *Rs*TSPO in the transition from aerobic to anaerobic growth suggests a role for TSPO under changing environmental conditions, which may correlate with the observed effects of distinct wavelengths of light or composition of growth medium on *FdTSPO* expression levels.

Although salt has been previously shown to decrease chlorophyll and phycobiliprotein content in WT *F. diplosiphon* (Singh and Montgomery, 2013b), we observed specific salt-dependent phenotypes in a Δ*FdTSPO* mutant relative to WT (**Figure 4**). Relatedly, a light-dependent salt susceptibility was reported for a *TSPO* mutant in *P. patens* when the mutant strain was switched from growth in the dark to a light/dark cycle (Frank et al., 2007). A lack of *Pp*TSPO1 under high salt conditions resulted in shrinking of gametophores and protonema filaments, and ultimately in cell death (Frank et al., 2007). Previous studies with salt treatment also reported an upregulation of TSPO at both the transcriptional and protein level in plants (Frank et al., 2007; Guillaumot et al., 2009; Balsemão-Pires et al., 2011) and at the transcriptional level in *Pseudomonas* (Leneveu-Jenvrin et al., 2015). In *A. thaliana*, an *AtTSPO* overexpression line exhibited a growth deficiency under salt stress compared to WT and a mutant line (Guillaumot et al., 2009). By comparison, salt-dependent bleaching was observed in *PpTSPO* mutant lines, but not WT (Frank et al., 2007). An elevated upregulation of stress marker genes, including drought responsive genes, was observed in the *AtTSPO* knock out line compared to the WT in response to salt (Balsemão-Pires et al., 2011). Thus, although correlated with salt-dependent responses in several photosynthetic organisms, the specific role for TSPO in response to stress can differ in distinct organisms.

Salt-stressed cyanobacterial cells have been reported to exhibit higher levels of ROS (Srivastava et al., 2005), reduced chlorophyll and phycobiliprotein content (Srivastava et al., 2008; Singh and Montgomery, 2013b), decreased oxygen evolution, decreased carbon fixation, and increased respiration (Srivastava et al., 2008). The lowered ROS content of the Δ*FdTSPO* mutant in response to salt could be a consequence of lowered pigment content in the mutant leading to decreased light absorption, possibly causing less ROS to be formed and thereby improving fitness under salt stress. On the other hand increased levels of superoxide dismutase and catalase upon salt stress have been reported in cyanobacteria (Srivastava et al., 2008; Srivastava, 2010). In plants, catalase is inhibited under salt stress (Foyer et al., 1994), whereas overexpression of *E. coli* catalase in *Synechococcus* infers resistance to salt stress (Kaku et al., 2000). It is therefore possible that *Fd*TSPO influences ROS levels under salt stress through impacting cellular ROS detoxification capacity, which correlates with ROS-detoxification mechanisms (Busch and Montgomery, 2015).

The potential function of TSPO in the salt stress response in plants has become clearer with the recent finding of a physical interaction of *At*TSPO with an aquaporin (Hachez et al., 2014). These findings suggest a role of *At*TSPO in negative posttranscriptional regulation of aquaporin in the early stress response that is associated with a prevention of water loss upon salt stress (Hachez et al., 2014). Transient upregulation of *TSPO* early during exponential phase growth of *Pseudomonas* in response to salt treatment also has been noted (Leneveu-Jenvrin et al., 2015). These results are consistent with our observations that place TSPO in the early stress response and fine-tuning of the regulation of this response in *F. diplosiphon*.

Our observations suggest an involvement of *Fd*TSPO in abiotic stress responses. In the absence of *Fd*TSPO, parts of the early stress response involving energy-requiring, *Fd*TSPO-dependent acclimation processes are likely impaired resulting in a transient advantage for the mutant cell under salt stress. By contrast, the absence of *Fd*TSPO under osmotic stress impairs the ability of cells to grow and survive under GL. In the presence of extreme oxidative stress, as in the case of externally applied MV, a lack of *Fd*TSPO accompanied by a misregulated stress response would result in higher ROS generation and elevated cell death. The greater effect of MV-induced ROS generation under GL compared to RL is likely due to an already upregulated oxidative stress response machinery under RL compared to GL (Pattanaik et al., 2014). Dysregulation of tetrapyrrole metabolism as well as iron limitation might contribute to this increase in ROS (Kruse et al., 1995; Michel and Pistorius, 2004; Latifi et al., 2005; Jung et al., 2008; Pattanaik et al., 2014). We recently demonstrated that sensory light-perception and iron availability are intertwined with ROS formation and the oxidative stress response in *F. diplosiphon* (Pattanaik et al., 2014), processes in which *Fd*TSPO might be involved or through which it is regulated (**Table 1**).

The Δ*FdTSPO* mutant exhibited altered light-dependent regulation of cellular morphology compared to WT (**Figure 8**). The morphology phenotype observed for the Δ*FdTSPO* mutant could be due to perturbations in the light-quality mediated regulation of morphology (Bordowitz and Montgomery, 2008), especially given light quality-dependent regulation of *FdTSPO* mRNA accumulation. On the other hand, as TSPO is a predicted membrane protein, we cannot exclude the possibility that a lack of *Fd*TSPO causes an indirect morphological change.

We found TSPO from *F. diplosiphon* to be involved in stress-related processes, specifically salt, osmotic and oxidative stresses. TSPO has been implicated in stress responses in photosynthetic organisms before (Frank et al., 2007; Balsemão-Pires et al., 2011). We found a unique connection to light-quality-related processes specific to a chromatically acclimating cyanobacterium, including higher resistance to salt stress under RL compared to GL, and higher susceptibility to osmotic stress and MV-induced oxidative stress under GL compared to RL in the mutant. Thus, TSPO seems to share certain functionalities across kingdoms, while having evolved species-specific functions.

## Author Contributions

AB and BM conceived and designed experiments, and AB conducted experiments. AB and BM analyzed data and wrote and edited the paper.

## Conflict of Interest Statement

The authors declare that the research was conducted in the absence of any commercial or financial relationships that could be construed as a potential conflict of interest.
