# Supplementary material for: The Tryptophan-Rich Sensory Protein (TSPO) is Involved in Stress-Related and Light-Dependent Processes in the Cyanobacterium Fremyella diplosiphon
Source: Front Microbiol. 2015 Dec 14;6:1393. doi: 10.3389/fmicb.2015.01393 (PMC4677103; doi:10.3389/fmicb.2015.01393)

Supplementary Material

The tryptophan-rich sensory protein (TSPO) is involved in stress-related and light-dependent processes in the cyanobacterium *Fremyella diplosiphon*

Andrea W. U. Busch and Beronda L. Montgomery*

*** Correspondence:** Beronda L. Montgomery: montg133@msu.edu

1 Supplementary Data

Supplementary Figure 1: Internal iron content analysis in *F. diplosiphon* SF33 wild type (WT) and Δ*FdTSPO* knockout mutant strains. The intracellular iron content was determined for cells grown in medium supplemented with (+Fe) or lacking (-Fe) iron under green light (GL) or red light (RL) and measured employing inductively coupled plasma optical emission spectrometry (ICP- OES). Bars represent means (±SD) for two replicate samples.

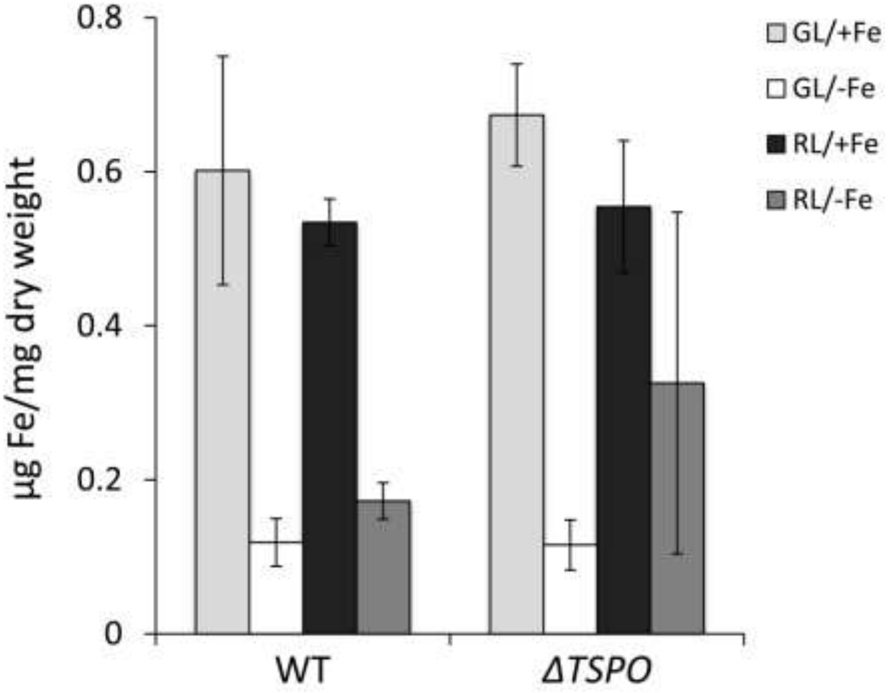

Supplementary Figure 2: Whole cell spectra of wild type (WT) and Δ*FdTSPO* cells. Cells in exponential phase growing under green light (GL) or red light (RL) were diluted to OD_{750} = 0.1 and the absorbance measured between 350 nm and 750 nm in 10 nm intervals for three independent cultures for each condition.

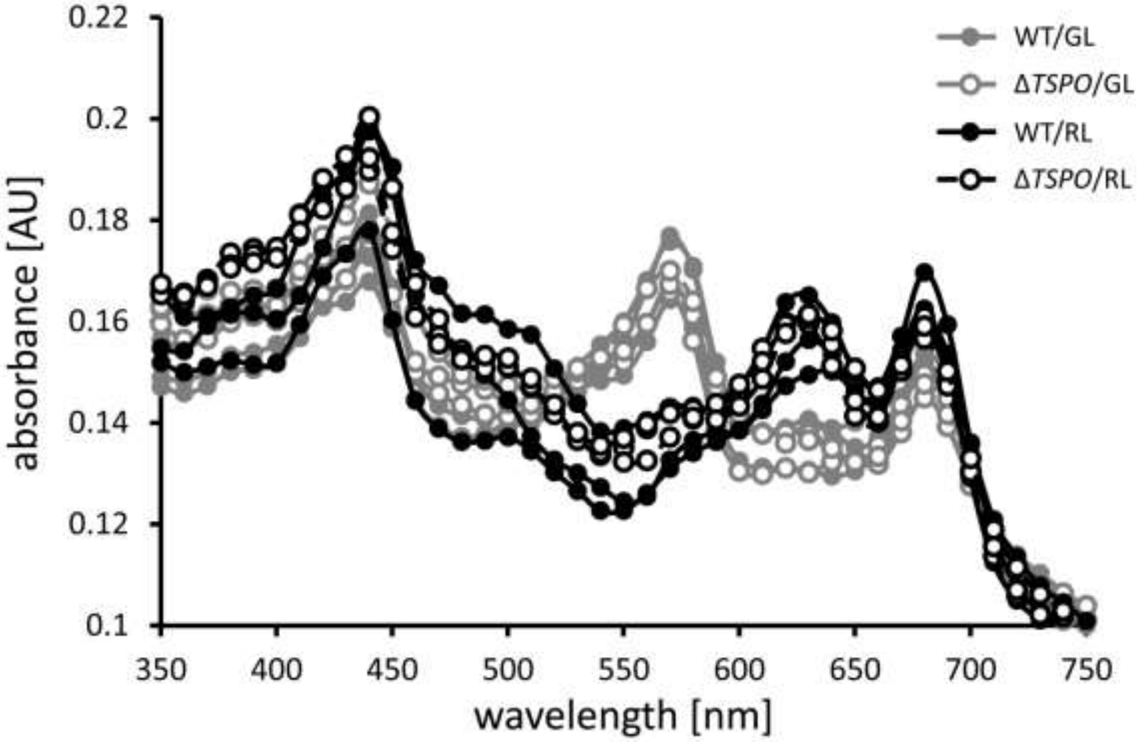

Supplementary Figure 3: Growth of Δ*FdTSPO* mutant compared to wild type (WT) under standard condition. Cells were grown at ~10 μmol m^{-2} s^{-1} under red light (RL) or green light (GL) and growth was monitored at OD_{750} over 11 days.

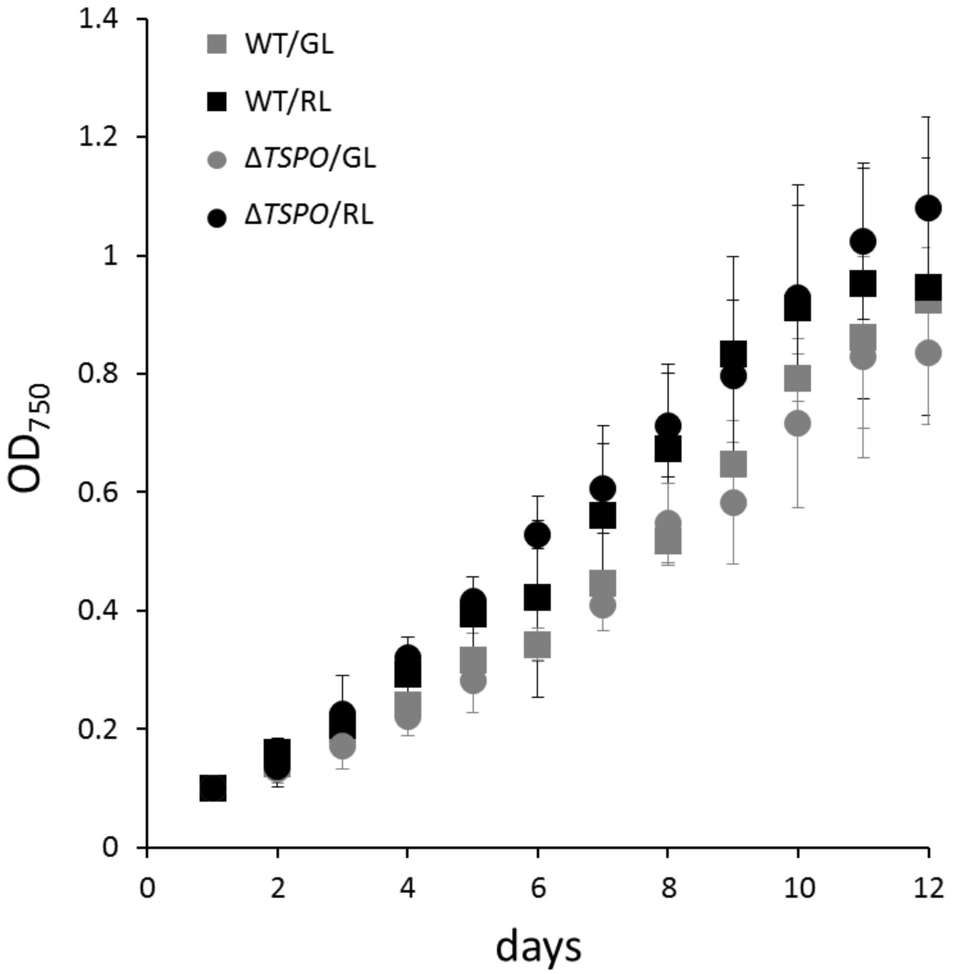

Supplementary Figure 4: Growth of complemented Δ*FdTSPO* mutant [np*TSPO*(Δ*TSPO*)] compared to wild type (WT) transformed with empty pPL2.7 vector [pPL2.7(WT)] under low-light condition. Cells were grown at ~10 μmol m^{-2} s^{-1} under red light (RL) or green light (GL) and growth was monitored at OD_{750} over 6 days. The Δ*FdTSPO* mutant strain complemented with a plasmid-originated WT copy of *FdTSPO* driven by its native promoter [i.e., npTSPO (Δ*TSPO*)] shows significantly reduced growth after 3 d (*p<0.01) and 6 d (**p<0.05), as determined by ANOVA and Tukey test, of growth in BG11/HEPES supplemented with 200 mM NaCl and 12.5 μg/ml kanamycin in RL.

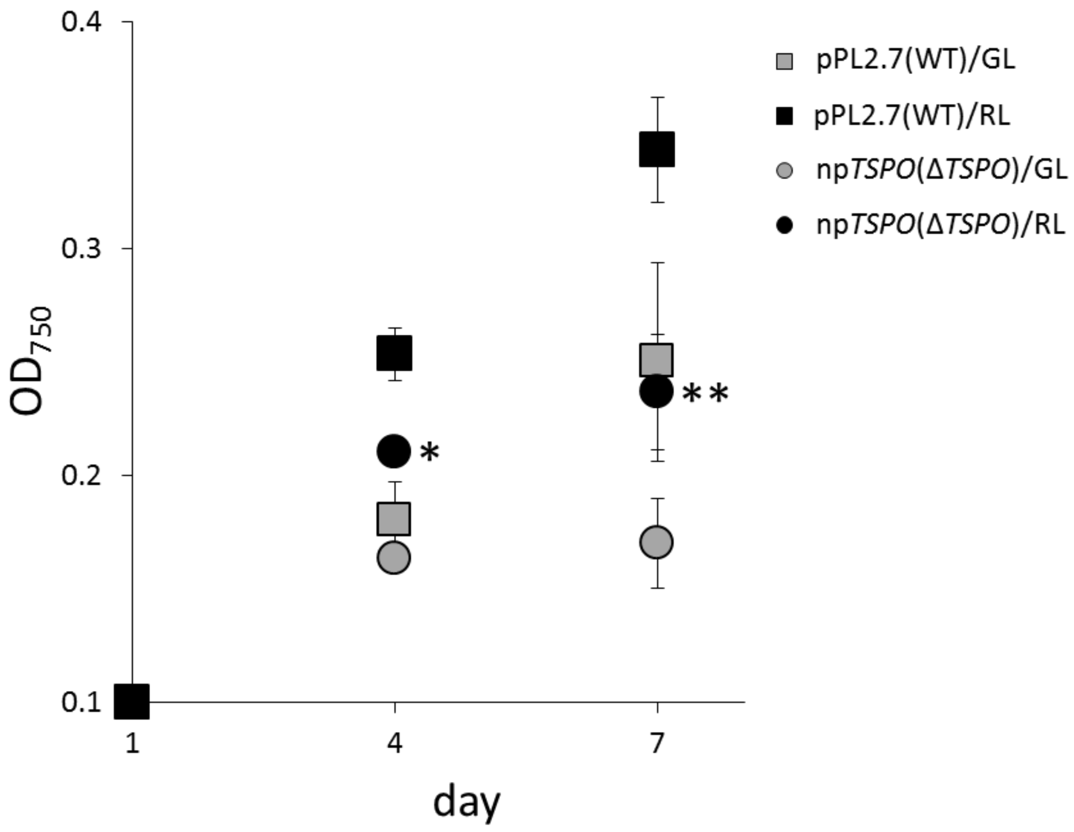

Supplement: Supplementary file 1 [file Presentation_1.PDF]
